# Supplementary material for: Distinctive phases and variability of vibration-induced postural reactions highlighted by center of pressure analysis
Source: PLoS One. 2023 Jan 23;18(1):e0280835. doi: 10.1371/journal.pone.0280835 (PMC9870114; doi:10.1371/journal.pone.0280835)
Supplement: Attachment — Submitted filename: Response to reviewers.docx [file pone.0280835.s002.docx]

**Point by point reply to the Reviewer’s comments**

*Dear Editorial team and Reviewer,*

*Thank you for giving us the opportunity to improve our manuscript. All comments from the Reviewer have been answered below. Whenever necessary, improvements were made in the manuscript and in the Table, identified in red. We thank the Reviewer and Editors for their time and precious help regarding our manuscript.*

*Sincerely*

**Louis-David Beaulieu, PhD, PT**

Associate Professor - FRQS Research Scholar Junior 1

Director, BioNR Research Lab

University of Quebec in Chicoutimi

**Response to Reviewer #1 comments:**

**General comments**

***Comment #1:*** *The manuscript explores the analysis of postural responses following tendon vibrations, where responses are separated into distinct phases. As hypothesised, stronger postural responses are present during the early phase of vibration compared to the later ‘stabilisation’ phase, supporting the need for analysis to be separated into these distinct phases, as opposed to ‘whole-trial’ analyses. Overall, the justification for the research and methodological approach to answer the specific research question seem appropriate. However, there are a number of major/minor comments that remain. Please see my questions, comments, and suggestions for each section below.*

**Answer**: We sincerely thank the Reviewer for their constructive and helpful suggestions for improving our manuscript, which are addressed below. All changes in the manuscript are identified in red in the text.

**Comments per section**

**Title**

***Comment #2:*** *Firstly, the title is too long and is “unappealing” – please re-work.*

**Answer**: The title was reduced as follow:

"Distinctive phases and variability of vibration-induced postural reactions highlighted by center of pressure analysis"

**Abstract**

***Comment #3:*** *Line 39 – Can you be more specific in the results section of the abstract? Which COP variables were higher and what was the magnitude of the difference (i.e, add in some of the data)?*

**Answer:** The requested data (most important findings only to avoid too much words in the Abstract) have been added to the result section of the Abstract.

“COP variables were generally higher during the early 2 s phase compared to the later 8 s phase for both TIB [mean difference between 8 s – 2 s phases: Amplitude AP = -1.11 ± 1.14 cm during VIB and -2.99 ± 1.31 during post-VIB; Velocity AP = -1.17 ± 0.86 cm/s during VIB and -3.13 ± 1.31 cm/s during post-VIB] and ACH tendons [Amplitude AP = -0.37 ± 0.98 cm during VIB and -3.41 ± 1.20 during post-VIB; Velocity AP = -0.31 ± 0.59 cm/s during VIB and -3.89 ± 1.52 cm/s during post-VIB].”

**Introduction**

***Comment #4:*** *Line 56 – should be “decade”, instead of “decades”.*

***Comment #5:*** *Line 57 – spelling mistake – should be “ageing”, instead of “aging”*

***Comment #6:*** *Line 75 – I would suggest replacing “, that consisted” with “and consisting” to make it clear that the two points are related to the 2-sec period, before you discuss the 8-sec period.*

***Comment #7:*** *Line 77 – should be “patterns” instead of “pattern”*

**Answer:** Thank you for these suggestions about spelling errors. Changes were made accordingly in the manuscript.

***Comment #8:*** *Line 98-102 – what were your hypotheses for the variation data? Please include.*

**Answer:** The hypotheses regarding data variability was included in the text as follows:

“Considering our previous research [2], we hypothesized that variability of VIB-PR will be lower with longer measurement periods (i.e. whole 10 s vs. last 8 s vs. early 2 s). Indeed, reliability metrics in our previous work systematically improved when increasing time-windows of measurement.”

**Method**

***Comment #9:*** *I do have one general question about the method adopted within this paper. You state in your introduction that VIB can simulate postural responses similar to falling etc. – if this is the case, why do you perform the vibration for such a long period? If this were to be related to falls, the stimulus and response to a fall would be almost instantaneous, so why would you be interested in seeing the response to the vibrations over longer periods than this?*

**Answer:** Thank you for this very relevant question. In fact, most previous studies used very short stimulation durations (about 3 s and less) which is more than enough to study sensory processing and postural reactions in response to challenging situations presenting a risk of falling. As seen in our study and others before, COP-related measures highlight that in normal individuals a brief and instantaneous reaction occurs to counteract the false signal of falling originating from VIB stimulation. However, using longer simulation durations (such as 10 s in the present study or up to 20 s and more in others [1]) does not lead to falling since other sensory sources (feet soles mechanoreceptors, vestibular system, muscle spindles from other muscles, etc.) are eventually identified by the CNS as more reliable than the one originating from the vibrated muscles (in accordance with theory of sensory re-weighting). In the present work we provided evidence and argued that these different phases of quick postural reaction early on, followed by a later re-stabilization underlies different mechanisms and can only be highlighted when using longer stimulation durations. So far, no previous study tried to identify and separately analyze these different patterns. They either chose too short durations to observe signs of sensory re-weighting, or analyzed their data as a whole (i.e. one single amplitude or velocity measure for the whole period of vibration) therefore missing the dynamic evolution of COP position and postural reactions. These ideas are extensively discussed in section 4.1 and on line 399 (limitation), but we agree that more details were necessary in the Methods:

“Vibration was activated at 80 Hz frequency and 1mm amplitude [2, 3]. There is no published guideline for the best duration of vibration to be used for eliciting postural reactions. We chose 10 s based on our previous study which was able to induce strong and reliable reactions and was long enough for most participants to reach the re-stabilisation phase [4].”

***Comment #10:*** *Line 107 – “and” should be “or” to detail the fact that participants had no history of disorders or injuries, as opposed to the two co-occurring.*

**Answer:** “and” was changed for “or” in the text.

***Comment #11:*** *Line 110-111 – can you put the units with the corresponding data, as opposed to the descriptor?*

**Answer:** We modified in the text as follows:

“Participants’ characteristics were (mean ± standard deviation): age = 24.8 ± 2.4 years; height = 171.4 ± 9.4 cm; body mass = 73.7 ± 16.0 kg”

***Comment #12:*** *Line 110 – this is body mass (kg), not body weight (N).*

**Answer:** Thank you**.** This was corrected (cf. response to Comment #11).

***Comment #13:*** *Line 117 – “were performed” as opposed to “were done”.*

**Answer:** Done.

***Comment #14:*** *Line 117 – was the same rest period given between conditions?*

**Answer:** Thank you for having pointing this out. Rests between conditions were slightly longer (5min) since we had to change the position of the vibrators. Participants were asked to sit on a chair while we replaced the vibrators. More details were added to the manuscript:

“Five trials per condition were done (with 1 min rest between trials and approximately 5 min rest between conditions while participants sat on a chair and vibrators were repositioned over the next targeted tendons).”

***Comment #15:*** *Line 118 – what do you mean by along the body? Arms by their sides? Hanging freely?*

**Answer:** In fact, arms were by their sides along the body (now specified in the text).

***Comment #16:*** *Line 119-120 – What was the feet position?*

**Answer:** The following information was added to the text **:**

**“**Precisely, participants were first asked to position their feet hip-width apart with their heels aligned on tape markers placed posteriorly along the X axis. Then, big toes were aligned on the Y axis and tape markers were added along the 5^th^ metatarsal. Care was taken to ensure that this standardized position was kept throughout the experiment.”

***Comment #17:*** *Line 121 – can you be more specific on the locations of the vibration devices? How did you standardise these between participants?*

**Answer:** This is a very interesting comment since (to our knowledge) no previous study tested different positioning of vibrators or proposed guidelines to ensure best efficiency and reproducibility of VIB-PR. The targeted ankle tendons are easily identifiable through palpation and observation, which might explain why details are often lacking in similar studies about the vibrator’s position. We added in the manuscript the following supplementary information how we proceed in our Lab (with acceptable reliability – cf [4]):

“Tendons were identified by palpation and slight contraction (ACH tendon located proximally to the calcaneal tuberosity and TIB on the anteromedial surface of the ankle above the talus and near the medial malleolus). Each vibrator comes with a small depression at its center in which the tendon is placed for improving comfort and stability of the device. For both ACH and TIB conditions, vibrators were localised approximately at the level of medial and lateral malleoli.”

***Comment #18:*** *Line 130 – can you be more specific? What were the linear COP variables?*

**Answer:** This was added in the text as follows: “…were used to calculate the selected linear COP (Amplitude and Velocity) variables”.

***Comment #19:*** *Line 140 – “(REF)”? Is there supposed to be a supporting reference here? Please either add the appropriate reference, or remove.*

**Answer:** Thank you for having found this omission. The appropriate reference [2] was added in the text.

***Comment #20:*** *Line 161 – what pairwise comparisons did you run?*

**Answer:** We used the paired samples t-test. This has been specified in the manuscript.

***Comment #21:*** *Line 168 – replace “having” with “with”*

**Answer:** Done!

**Results**

***Comment #22:*** *Throughout this whole section, you hardly report any actual results. Throughout, please provide more detail, including actual data (measured values, group mean differences etc.), and the results of your statistical tests.*

*In my opinion, figures 3 and 4, where you present variability data, should not be presented as bar charts – even where data are normally distributed. Bar charts arbitrarily assign importance to the height of the bar only. Whilst this is obviously an important factor, what it fails to show is how the difference between the means compares to the range of observed values. Using either a dot-plot, or combining a dot-plot with your bar chart would better display this data and paint a “fuller” picture of this variability data.*

**Answer:** We agree with the Reviewer that giving more data would be highly relevant. To keep the text’s readability, we decided to add a new Table (Table 2) which gives all statistics, differences and effect sizes between time-windows (i.e. Whole 10 s – First 2 s; Whole 10 s – Last 8 s; Last 8s – First 2 s) for the two conditions of VIB and the two periods (VIB and post-VIB). If the Reviewer and Editors prefer that these details are presented in the text instead of a new Table we will make the change with pleasure.

The following changes were made in the Methods and Results:

“The level of statistical significance was set at p < 0.05 and effect sizes (Cohen’s d ES) were calculated (G*Power 3.1 Software) whenever relevant to give a perspective about the magnitude of the effects (large if >0.8, moderate if >0.5, small if >0.2 and null if <0.2).”

***“***3.2 Differences between *First 2s*, *Last 8 s* and *Whole 10 s* time-windows

Table 1 presents mean ± SD of COP variables selected for the present study and Table 2 details results for statistical comparisons between time-windows for the tested conditions of vibration, including mean differences and Cohen’s d effect sizes. ANOVA revealed significant effects (p < 0.05) for all comparisons between time-windows during VIB and Post-VIB periods. In general, pair-wise comparisons between the three time-windows reached the level of statistical significance and had moderate to high effect sizes, with similar findings observed for VIB and Post-VIB periods. *Amplitude AP* was highest during the *Whole 10 s* block, followed by *First 2 s* and *Last 8s* (except during VIB of ACH which was not different between *First 2 s* and *Last 8 s* time-windows). Conversely, *Velocity AP* had highest values during the *First 2 s*, followed by *Whole 10 s* and lowest values obtained for *Last 8 s*.

*Insert Tables 1 and 2 near here*”

However, the suggestion about Figures 3 and 4 (bar charts vs dot-plot) is not possible with SEMeas, SD and CV data since these variability metrics provide a unique number for the whole group. Bar charts in Figures 3 and 4 are not means but rather the exact (and only) data calculated. SEMeas for example computes the within-subject error value between tests and propose a unique “standard” error that should encompass the expected variations of scores between tests in any individual presenting with the same characteristics than our sample. If we did not properly understand the Reviewer’s comment, we would be happy to further discuss this issue and make appropriate changes if needed in a future revision.

***Comment #23:*** *Line 187 – you did not define BL – I assumed it means baseline.*

**Answer:** Indeed. baseline (BL) was added.

***Comment #24:*** *Line 188-189 – is this not the same result you present in line 185? If so, please remove. If not, can you make it clear what is different?*

**Answer:** We agree that this sentence lacks clarity. It is indeed a different result than what was presented above. The small COP displacement described on lines 188-189 occurs during the first 0.5 s after VIB start, as identified with the red circle below:

Possible interpretations for this finding are explored in the Discussion (lines 285-295 in the original version of the manuscript). To improve the clarity of the sentence, we made the following changes:

“Furthermore, the early reaction for both VIB and Post-VIB periods started with a small COP displacement in the reverse direction observable during the first milliseconds after VIB start and cessation (Figure 1 A & B). For example, Figure 1 A shows that when VIB is activated, COP position in fact goes posteriorly for about 0.5 s before quickly moving anteriorly in the following seconds.”

***Comment #25:*** *Line 204 – do you mean* *p < 0.05?*

**Answer:** Indeed, (p < 0.05) was added in the text.

***Comment #26:*** *Line 205-207 – here is an example where the individual significance values would be useful.*

***Comment #27:*** *Line 207 -211 – can these differences be indicated in the table to show exactly which comparisons were significant and to what level?*

**Answer:** Thank you for the suggestion. As described in response to Comment #22 all results are now presented in a new Table to facilitate the readability of the results.

***Comment #28:*** *Line 216 – “p = 0.11” instead of “p value of 0.11)”.*

**Answer:** This was corrected in the text.

***Comment #29:*** *Line 229-230 – they increased – but from what to what? Or by how much?*

**Answer:** More details were added to this section:

“Conversely, more contrasts were found between time-windows during post-VIB periods. For *Amplitude AP*, *First 2* *s* and *Whole 10 s* windows were about 3.0 times higher than *Last 8 s* for both TIB and ACH conditions. For *Velocity AP*, only the *First 2 s* remained higher, with scores 2.7-4.5 times higher than *Last 8 s* and *Whole 10 s* time-blocks. When comparing VIB and post-VIB periods, group SD tended to increase at post-VIB for *First 2 s* of TIB and ACH conditions (ranging 1.5-2.2 times higher). Conversely, a 2-fold decrease (ranging 1.9-2.1) of group SD was found for *Last 8 s* during post-VIB vs. VIB periods. No clear pattern was found for the *Whole 10 s* which either remained similar, increased or decreased at post-VIB compared to VIB periods.”

**Discussion**

***Comment #30:*** *Line 242 – In the second and third sentences of your first paragraph, can you provide a statement of the importance or relevance of these findings?*

**Answer:** Thank you for this suggestion. The following changes were made:

“Results from the present study confirmed our primary hypotheses since spatiotemporal analyses of COP displacements mostly revealed stronger postural reactions during the early 2 s phase, whereas the later 8 s phase was characterized, in general, by a progressive slowing of COP displacements. Most within- and between-subject variability scores for the selected COP variables were below 30% and using three instead of five trials had no impact on variability, hence advancing our understanding of the proposed approach from methodological and metrological standpoints. VIB-PR patterns for the early and later phases were quite similar within a same person (Figure 2), but distinctive behaviors were observed between individuals during the later phase, suggesting that this VIB-PR phase could be more sensitive to discriminate individuals presenting with different patterns of reactions to a sensory disturbance. Overall, our study highlights the relevance of identifying and separately analyzing distinct phases within VIB-PR patterns, as well as characterizing these patterns at the individual level.”

***Comment #31:*** *Line 263 – can is a strong statement to make when you do not know for sure. Please use “may” instead.*

**Answer:** We agree. This was replaced by “may” in the text.

***Comment #32:*** *Line 275 – reference is in different format to the remainders. Please incorporate the reference number.*

**Answer:** Thank you. The reference was corrected “ Kavounoudias et al. [1].”

***Comment #33:*** *Line 301-303 – Is this truly the case? According to your figures, the average COP position changed by ~3cm, whereas the average adult foot would be nearer 25-30cm? It would have been nice to see a figure visualising where on the foot the COP is positioned. If you have the capacity to show such a figure with this data, I would recommend including, but I appreciate that it may not be possible with your available dataset (maybe something to consider if you perform similar work in the future).*

**Answer:** This is an important observation from the Reviewer, we unfortunately don’t have the capacity to provide such an image (but will clearly keep the idea for the future). However, the way the text was written could be misleading, hence possibly explaining the Reviewer’s comment. As nicely raised by the Reviewer, COP displacements were not as long as the length of the feet and therefore terms like “far up” might not be adequate. Still, for a COP position to move of 3-4 cm anteriorly for example, one would nearly reach its limits of stability if the foot sole remains in contact with the force plate (which was the case in our study). Therefore, even if COP moved anteriorly, pressure was still applied through the heels, but to a lower extent than through forefoots and toes. The mean COP excursion is therefore limited to a few centimeters even though the overall postural reaction can almost reach the limits of stability. If participants had raised parts of their feet (e.g. heels lifting during an anterior postural reaction) from the force plate we would have measured longer COP amplitudes but this did not happen. To avoid misleading readers, we re-phrased the following sentence:

“In our example of TIB vibration, the COP moves quickly from slightly posterior to its baseline position 0.5 s after VIB start to a more anterior position toward toes and forefoots as the plantar flexors contract to decelerate the forward movement of the center of mass.”

***Comment #34:*** *Line 314-316 – plastic adaptations indicate that there is some sort of permanent change. Is there evidence to suggest that this would be the case?*

**Answer:** Thank you for this comment. “plastic” was used here as a broad term encompassing both rapid (synaptic) and lasting (morphologic) changes that could occur in networks involved in sensorimotor processing and postural adaptations. In case of acute effects after short periods of stimulation like we used, we believe that any changes that lasted would likely be temporary and completely disappear after a few seconds/minutes which could be more logically ascribed to synaptic changes instead of morphologic. Of course, we did not use any measurement tools that would inform about potential mechanisms involved, so we did not want to expand further on this topic in the manuscript. Considering the Reviewer’s comment, we made the following changes:

“Mechanisms involved afterwards during the later phase are less evident to explain without additional neurophysiological outcome measures. Under continuous VIB, the postural control system would have to find a new equilibrated state through this contradicting multisensory information. Reaching a stabilized COP position during vibration would require complex sensorimotor control mechanisms leading to plastic (synaptic and/or morphologic) adaptations of spinal and brain circuitries involved in the processing of sensory afferents. The clear post-VIB effects found in the present study and others before [5, 6] are all suggestive signs of potential plastic adaptations within CNS networks involved in postural control. Future research should consider using neurophysiological tools (e.g. peripheral and central neurostimulation, electromyography, brain imagery) to further explore post-VIB effects and their underlying mechanisms.”

***Comment #35:*** *Line 317 – no previous definition of CNS – please write out in full.*

**Answer:** Done! Thank you

***Comment #36:*** *Line 329-332 – is this something you are looking to address in the future? If so, include as a future direction.*

**Answer:** The Reviewer is right; we are indeed interested in addressing this in the future. A statement was added:

“Since the present work focused on methodological considerations and did not include neurophysiological measures to uncover these mechanisms, we cannot propose reliable explanations to account for such between-subject variability. Future studies are underway using electromyography and peripheral/central neurostimulators to address this question. Nevertheless, our study underscores the relevance of an individually tailored analysis of VIB-PR behaviors which adds complementary knowledge to group analysis.”

***Comment #37:*** *Line 339 – what do you mean by anteroposterior plan? I assume this is a typo?*

**Answer:** Anteroposterior plan characterizes the vertical direction of the center of foot pressure displacement [2]

***REFERENCES CITED IN THE RESPONSE TO REVIEWER’S COMMENTs***

1.	Duclos NC, Maynard L, Barthelemy J, Mesure S. Postural stabilization during bilateral and unilateral vibration of ankle muscles in the sagittal and frontal planes. J Neuroeng Rehabil. 2014;11:130.

2.	Polonyova A, Hlavacka F. Human postural responses to different frequency vibrations of lower leg muscles. Physiol Res. 2001;50(4):405-10.

3.	Roll JP, Vedel JP. Kinaesthetic role of muscle afferents in man, studied by tendon vibration and microneurography. Experimental Brain Research. 1982;47(2):177-90.

4.	Kadri MA, Chevalier G, Mecheri H, Ngomo S, Lavalliere M, da Silva RA, et al. Time course and variability of tendinous vibration-induced postural reactions in forward and backward directions. J Electromyogr Kinesiol. 2020;51:102386.

5.	Wierzbicka MM, Gilhodes JC, Roll JP. Vibration-induced postural posteffects. J Neurophysiol. 1998;79(1):143-50.

6.	Duclos C, Roll R, Kavounoudias A, Roll JP, Forget R. Vibration-induced post-effects: a means to improve postural asymmetry in lower leg amputees? Gait Posture. 2007;26(4):595-602.
