# Supplementary material for: Distinctive phases and variability of vibration-induced postural reactions highlighted by center of pressure analysis
Source: PLoS One. 2023 Jan 23;18(1):e0280835. doi: 10.1371/journal.pone.0280835 (PMC9870114; doi:10.1371/journal.pone.0280835)
Supplement: Attachment — Submitted filename: Reviewer Comments.pdf [file pone.0280835.s001.pdf]

Review: Distinctive phases and intra/inter-individual variability of vibration-induced postural reactions highlighted by spatiotemporal analyses of center of pressure displacements

The manuscript explores the analysis of postural responses following tendon vibrations, where responses are separated into distinct phases. As hypothesised, stronger postural responses are present during the early phase of vibration compared to the later 'stabilisation' phase, supporting the need for analysis to be separated into these distinct phases, as opposed to 'whole-trial' analyses. Overall, the justification for the research and methodological approach to answer the specific research question seem appropriate. However, there are a number of major/minor comments that remain.

Please see my questions, comments, and suggestions for each section below.

Firstly, the title is too long and is "unappealing" – please re-work.

Abstract

Major comments:

No major comments - the abstract summarises the work clearly.

Minor comments:

Line 39 – Can you be more specific in the results section of the abstract? Which COP variables were higher and what was the magnitude of the difference (i.e, add in some of the data)?

Introduction

Major comments:

No major comments for this section. In general, the introduction provides a nice overview of the area and justification for the work.

Minor comments:

Line 56 – should be "decade", instead of "decades".

Line 57 – spelling mistake – should be "ageing", instead of "aging"

Line 75 – I would suggest replacing ", that consisted" with "and consisting" to make it clear that the two points are related to the 2-sec period, before you discuss the 8-sec period.

Line 77 – should be "patterns" instead of "pattern"

Line 98-102 – what were your hypotheses for the variation data? Please include.

Method

Major comments:

I do have one general question about the method adopted within this paper. You state in your introduction that VIB can simulate postural responses similar to falling etc. – if this is the case, why do you perform the vibration for such a long period? If this were to be related to falls, the stimulus and response to a fall would be almost instantaneous, so why would you be interested in seeing the response to the vibrations over longer periods than this?

Minor comments:

Line 107 – "and" should be "or" to detail the fact that participants had no history of disorders or injuries, as opposed to the two co-occurring.

Line 110-111 – can you put the units with the corresponding data, as opposed to the descriptor?

Line 110 – this is body mass (kg), not body weight (N).

Line 117 – "were performed" as opposed to "were done".

Line 117 – was the same rest period given between conditions?

Line 118 – what do you mean by along the body? Arms by their sides? Hanging freely?

Line 119-120 – What was the feet position?

Line 121 – can you be more specific on the locations of the vibration devices? How did you standardise these between participants?

Line 130 – can you be more specific? What were the linear COP variables?

Line 140 – "(REF)"? Is there supposed to be a supporting reference here? Please either add the appropriate reference, or remove.

Line 161 – what pairwise comparisons did you run?

Line 168 – replace "having" with "with"

Results

Major comments:

Throughout this whole section, you hardly report any actual results. Throughout, please provide more detail, including actual data (measured values, group mean differences etc.), and the results of your statistical tests.

In my opinion, figures 3 and 4, where you present variability data, should not be presented as bar charts – even where data are normally distributed. Bar charts arbitrarily assign importance to the height of the bar only. Whilst this is obviously an important factor, what it fails to show is how the difference between the means compares to the range of observed values. Using either a dot-plot, or combining a dot-plot with your bar chart would better display this data and paint a "fuller" picture of this variability data.

Minor comments:

Line 187 – you did not define BL – I assumed it means baseline.

Line 188-189 – is this not the same result you present in line 185? If so, please remove. If not, can you make it clear what is different?

Line 204 – do you mean $p < 0.05$?

Line 205-207 – here is an example where the individual significance values would be useful.

Line 207 -211 – can these differences be indicated in the table to show exactly which comparisons were significant and to what level?

Line 211 – "$p = 0.11$" instead of "p value of 0.11)".

Line 228 – again, very vague – what were the actual results?

Line 229-230 – they increased – but from what to what? Or by how much?

Discussion

Major comments:

No major comments for this section.

Minor comments:

Line 242 – In the second and third sentences of your first paragraph, can you provide a statement of the importance or relevance of these findings?

Line 258 – can is a strong statement to make when you do not know for sure. Please use "may" instead.

Line 270 – reference is in different format to the remainders. Please incorporate the reference number.

Line 301-303 – Is this truly the case? According to your figures, the average COP position changed by ~3cm, whereas the average adult foot would be nearer 25-30cm? It would have been nice to see a figure visualising where on the foot the COP is positioned. If you have the capacity to show such a figure with this data, I would recommend including, but I appreciate that it may not be possible with your available dataset (maybe something to consider if you perform similar work in the future).

Line 314-316 – plastic adaptations indicate that there is some sort of permanent change. Is there evidence to suggest that this would be the case?

Line 317 – no previous definition of CNS – please write out in full.

Line 329-332 – is this something you are looking to address in the future? If so, include as a future direction.

Line 339 – what do you mean by anteroposterior plan? I assume this is a typo?
